# Incorporating a Hands-On Device-Based Activity in a Human Factors Biomedical Engineering Course in Sub-Saharan Africa

**DOI:** 10.1007/s43683-024-00147-5

**Published:** 2024-05-06

**Authors:** Alick O Vweza, Sara Mehta, Matthew Wettergreen, Ann Saterbak

**Affiliations:** 1 Malawi University of Business and Applied Sciences, Blantyre, Malawi; 2 Department of Biomedical Engineering, Duke University, 101 Science Drive, Durham, NC 27708 USA; 3 Rice University, 6100 Main Street, Houston, TX 77005 USA

**Keywords:** Human factors, Device evaluation, Design, Biomedical devices, Engineering education in sub-Saharan Africa

## Abstract

A challenge in building the biomedical engineering human factors course at Malawi University of Business and Applied Sciences was integrating meaningful direct experiences with medical products. The instructor also noticed a significant gap between the topics in the course and their surrounding clinical context, a low-income setting. Recognizing that devices should be designed and evaluated in the context of the local users’ needs and situations, new hands-on modules were created and implemented in this BME human factors course. Students were asked to critically evaluate and make recommendations to improve the human factors aspects of the software and hardware of the IMPALA, a vital signs monitoring device developed for use in Malawi. Engaging with this medical device, students observed and understood many issues discussed in human factors, including the design of ports, controls, and other user interfaces. The collaboration between the course and the IMPALA project harnessed the local expertise of students to improve the design of a new patient monitoring system. Thus, the IMPALA project itself benefited from this collaboration. Second, students greatly benefited from applying the class concepts to the IMPALA. Students were engaged far more during the interactive components than during the lecture components. Many students successfully translated their knowledge on human factors to their final-year design project.

## Challenge Statement

Upon graduation, biomedical engineers are expected to enter a workforce where technology is applied to support the treatment and advancement of human health. Specifically, they are expected to have both knowledge and hands-on skills to innovate and design biomedical devices [1]. To meet this need, programs must keep pace with changing technology and engineering designs and actively participate in designing and implementing relevant medical device design projects [2, 3]. In the context of biomedical device design, the emphasis is often on designing, building, and testing the system's core functional components; however, often overlooked is the design of how humans interact with devices in real-world situations, a field referred to as human factors [4].

Human factors is the “science of the interrelationships between humans, their tools, and the environments in which they work” [5]. Safety and improvement efforts in healthcare are often thwarted by inadequate attention to human factors and ergonomics [5]. Careful attention to these topics during the design phase can improve provider safety, productivity, and efficiency, as well as satisfaction and daily experiences; thus, a human factors perspective is critical in biomedical engineering [5]. With continuous developments and advancements in healthcare, human factors engineering courses must also maintain current practices and be adaptive to industry developments [6]. Design-induced medical device error is a notable cause of patient mortality in both low-income and high-income settings [7]. Ultimately, what underscores the significance of human factors is that understanding how humans think and interact with technology increases our ability to design interactive systems that work as intended [8].

The need for appropriate consideration of human factors is even more important in low-income settings, where addressing health equity is a central issue for medical device designers. Too often, the context of users takes a backseat to the technological perspective of device design, while failing to consider human needs and the voice of the user [9–11]. Rodriguez et al. specifically place the responsibility for addressing these priorities in the hands of biomedical engineering designers; the authors highlight the many tools for applying a holistic approach, which includes not only the technical aspects of device design but also human-centered approaches [10]. Aranda et al. also offer suggestions for medical device designers and advocate for them to adopt a framework for understanding and capturing contextual information for designing medical technologies [9]. Burleson et al. provide such a framework, including classifications and applications of contextual factors, such as socio-cultural and environmental, to guide engineers through design processes [11].

Owing to the importance of understanding human interaction with products, Malawi University of Business and Applied Sciences (MUBAS) biomedical engineering (BME) department requires its students to take a Human Factors course (ELE-HFM-521). This relatively new course (first taught in 2021) is situated in the final year of a 5-year engineering bachelor's program. The course focuses on device design and how humans interact with medical devices. Compared to other courses in the degree program, the human factors course is not equation-heavy. Instead, it focuses on ideas and design principles. The department was motivated to add this course following an observation that graduates of the BME program were designing difficult-to-use medical devices (i.e., devices that needed more application of basic concepts from human factors).

A primary challenge in building the BME Human Factors course at MUBAS was integrating meaningful direct experiences with medical products. The instructor also noticed a significant gap in relating the indicative content (i.e., list of topics) of human factors courses with the surrounding clinical context and low-income setting. Recognizing that devices should be designed and evaluated in the context of the users’ needs and situations where the devices would be used, the instructor was surprised to find that many devices designed for low-income areas still used a high-income lens for human factors. Course content and syllabi published in the literature did not equip students with the understanding of medical devices that need to operate in clinics and hospitals in Malawi. Many examples of human factors for biomedical devices, both in textbooks and online, were set in Western contexts [6] and did not account for contextual elements such as intermittent power, high nurse-to-patient ratios, poor lighting, poor eyesight of medical personnel and patients, and relatively lower literacy levels. As a result, students entering the workforce were not as prepared to design products for the local users in Malawi and other sub-Saharan African countries. Thus, the second major challenge was to ensure that hands-on opportunities in this course were set in the context of Malawi, where graduates would be employed.

This paper describes and reflects on a novel project introduced and implemented in the Human Factors course (ELE-HFM-521) at MUBAS. This project allows students to apply their developing knowledge of human factors to support the ongoing design of a local medical device with the intention that students develop a clear understanding of the importance of the user and local context as well as human factors considerations when designing products.

### Novel Initiative

Prior educational work has shown that hands-on experiences in human factors education are natural and rewarding for students and educators [12]. At the outset of this educational innovation in the Human Factors course, there were two aims:

A. Transform the Human Factors course to include hands-on experiences that encourage the application of course materials by supporting the design of a medical product intended for local use, and
B. Instill an understanding of the importance of many dimensions of the user’s contextual environment [9] as central to the engineering design and evaluation of a novel medical product.

To achieve these aims, Dr. Alick Vweza at MUBAS used the IMPALA device as a lengthy case study for the Human Factors class. The IMPALA is a vital signs monitoring device intended for local hospital use. The IMPALA is being developed by the IMPALA Project [13], which is a consortium made up of Malawi and European partners including MUBAS. As described, the incorporation of the IMPALA device into the Human Factors course can serve a template for other programs, especially those in low-income settings, to develop context-appropriate materials and examples.

#### Background on Human Factors Course

The overarching aim of the Human Factors course (ELE-HFM-521) was to provide students with principles of human factors engineering essential for the design and safe use of medical devices, particularly for resource-constrained settings. The course syllabus emphasizes the design of medical devices for usability and reprocessing. The course’s learning outcomes are that at the end of the course, students should be able to do the following:

1. Describe the goals of human factors engineering in medical device design.
2. Apply usability evaluation methods in human factors engineering for medical devices.
3. Use human factors engineering methods to design medical device controls, displays, and instruction manuals.
4. Generate user interface requirements to design safe, effective, usable, and satisfying medical products.
5. Discuss the importance of medical device reprocessing as a human factors engineering issue.

Topics covered in the course include the following:

1. Human factors in medical device design: Medical errors and medical devices; goals and benefits of human factors engineering in the design of medical devices.
2. Usability evaluation methods: Usability inspection and testing; heuristic and cognitive walkthrough; common measurables in usability testing; scenarios and tasks; Poisson model for the number of usability issues; quantitative task analysis methods.
3. Medical device use errors: Use errors and their causes and consequences; root cause analysis; medical device design for errors.
4. Design of controls and displays: Designing physical and digital controls; control coding, movements, size and shape, feedback, placement, and touchscreen considerations; visual and auditory displays and alarms; human–computer interaction; user interface requirement generation.
5. Reusable medical devices, reprocessing, and design for maintenance: Reusable medical devices and medical device design; human factors considerations for medical device reprocessing and designing for maintenance; reprocessing optimization.

Textbooks by Branaghan et al. [14] and Wiklund et al. [15] served as references. The IMPALA case study device was used throughout the course to reinforce a practical understanding of the content.

#### Integration of the IMPALA Project

Currently, in low-income countries, there is a shortage of medical equipment. In 2017, the World Health Organization estimated that 50% to 80% of medical equipment in low-income countries needs to be fixed, creating a barrier to the ability of the health system to deliver health services to patients [16]. Others have suggested that medical equipment should be designed for this environment rather than shipping decommissioned medical equipment from high-income countries to Malawi and other low-income countries [17, 18].

**Fig. 1:**
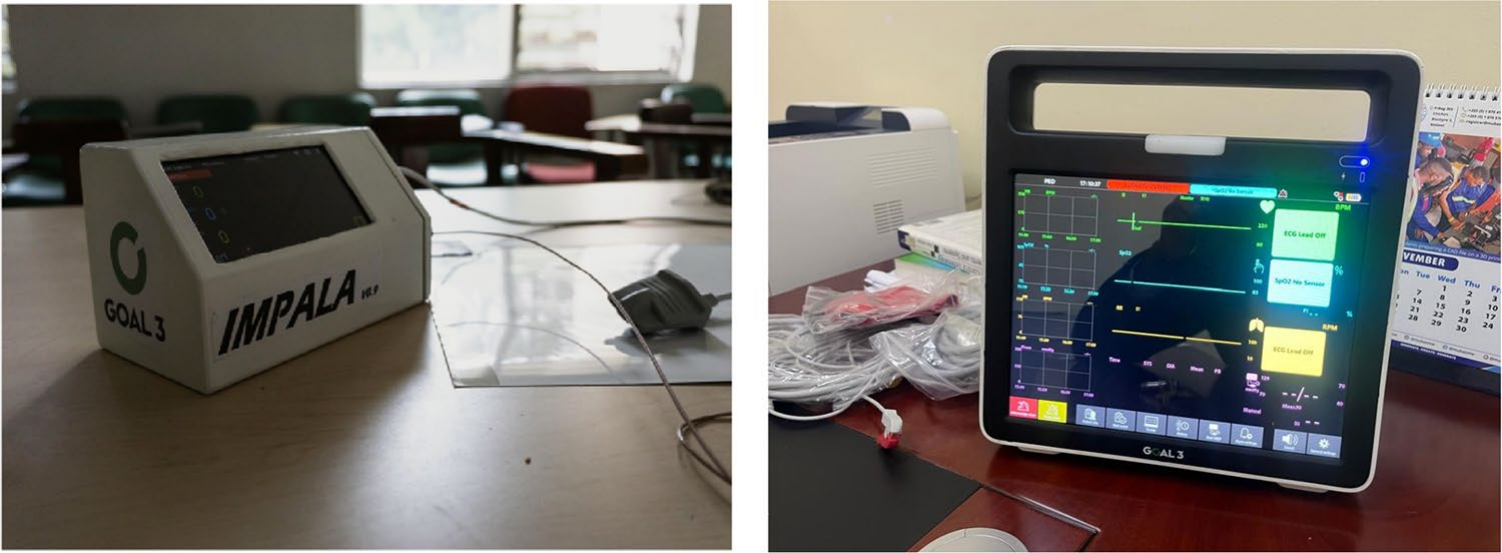
The IMPALA device [projectimpala.org] [13]. An early (at left) and late (at right) prototype of the IMPALA device. Dr. Alick Vweza used this example of the medical device development process to teach students the rigorous methods and revisions a medical device goes through, from concept to clinical trials to market.

The IMPALA project is an international partnership involving several Malawian and European institutions with a clear goal to improve healthcare delivery in local low-income settings. Specifically, the IMPALA project is developing an affordable, durable, and user-friendly vital signs monitoring system (Fig. 1) that is simple yet effective in facilitating healthcare workers to detect critical conditions early [13]. Monitoring vital signs, including heart rate, respiratory rate, etc., is necessary and standard care in a clinical environment. Monitoring systems used in high-income settings are often too expensive and incompatible with healthcare systems in low-income settings; thus, these monitors cannot be applied universally. Dr. Vweza and other engineering faculty at MUBAS and their European partners have been testing this product since 2022.

When used in the Human Factors course, the software and hardware of the IMPALA project were explored during the usability testing phase. Therefore, inherent in this project was the need to test the system and suggest changes that could improve human factors. Dr. Alick Vweza recognized that the class members could facilitate aspects of troubleshooting and device design. Engaging with this medical device, students could observe and understand many issues discussed in the Human Factors course, including the design of ports, controls, and other user interfaces. A sample lesson on User Interface Requirements for Medical Devices is given in the Appendix.

#### Integration of IMPALA Using the ICAP Framework

Integrating the IMPALA into the Human Factors course was completed using best practices in engineering and medical education [19–21]. Specifically, the instructor used the ICAP framework to guide the design of activities [22]. The ICAP framework is an active learning-based theoretical framework based on cognitive science [23]. It posits four modes of cognitive engagement: Interactive, Constructive, Active, and Passive. Previous research has found that students learn more when involved in active learning activities, such as self-study and question generation, before discussions [23]. The ICAP hypothesis, in particular, predicts that as students become more engaged with the teaching materials, from passive to active to constructive to interactive, their mastery of the material will increase. Driven by this framework, Dr. Alick Vweza changed ELE-HFM-521 to constructively engage with students to generate specific recommendations for the IMPALA and encourage interaction between students when engaging with the IMPALA device.

Each class included a lecture portion. Dr. Alick Vweza additionally integrated interaction with the IMPALA monitoring system for, on average, 20–30 min every class period (~ 25% of class time). When working with the IMPALA, he randomly divided the class in half. One-half of the students would serve as the ‘cognitive walkthrough’ team responsible for formulating questions and tasks on the user interface and their evaluation criteria (Table 1, columns A, B). The other half of the class would physically work with the IMPALA to encounter usability errors and reflect on the design of the device; this half would then try to address the usability issues by giving design recommendations (Table 1, columns C, D). Dr. Vweza instructed both teams to explore the context in which the device would be used, including local clinics and hospitals, where many students had already completed internships. As a result, students considered contextual considerations such as low lighting, intermittent power, high numbers of distractions, poor eyesight, etc. This regular activity during class was both constructive and interactive. Specifically, students worked in teams to construct new knowledge regarding the strengths, weaknesses, and recommendations for changing the device's design. Additionally, this work was done in a collaborative, peer learning environment, making the activity highly interactive.

**Table 1:** Example workflows in class are shown as rows in the table

A. Task	B. Evaluation criteria	C. Challenges identified	D. Recommendations made	E. Concepts from class
Task 1	Data entry efficiency	Placement of pop-up menu for entering patient information: The menu to start a pop-up for entering patient information was too close to the edge (i.e., too little room between the screen and the external casing).	Move the pop-up menu to a more user-friendly location to allow for quicker entering of patient information.	Design of displays, movements: touchscreen considerations, human–computer interaction, user interface requirement generation. (Topic 4)
Task 2	Satisfying user expectation; accessibility	Placement of ports: The ports for the sensors are placed on the left-hand side of the device, which makes it awkward when connecting the sensors for most right-handed people.	Place the ports on the right-hand side of device, while considering developing models for both right- and left-handed users.	Design of physical and digital controls: movements, size and shape, feedback, and placement. (Topic 4)
Task 3	Efficiency; accessibility	Access history of a patient: The time measured to retrieve patient history data exceeded the expected value.	Redesign the series of buttons/steps required to retrieve patient data more quickly.	Cognitive walkthrough, testing large numbers of users, use, and time of task. (Topics 2, 4)
Task 4	Error prevention and handling; satisfying user expectation; accessibility	Unexpected shutdown of device: The power ON/OFF button switches off the device without confirmation when long-pressed instead of bringing a pop-up menu for confirmation by the user if they want to switch the device off. This results in unintentionally switching off the device.	Redesign the software controller of the power ON/OFF button.	Design of controls and displays: Designing physical and digital controls; control coding, movements, size and shape, feedback, placement, and touchscreen considerations; medical device use errors. (Topics 3, 4)
Task 5	Satisfying user expectations	Battery life: The battery lasts about 5 hours when the screen is below normal brightness but reduces considerably to about 3 hours when brightness is increased. Since the device is designed for settings with unreliable mains power, the battery lifetime is not long enough for sustained patient monitoring as expected in the settings with power issues.	Increase the battery capacity to at least 6 hours under normal screen brightness, either through using a higher capacity battery or optimizing the hardware/software or both.	Design of controls and displays: Designing physical and digital controls; touchscreen considerations. (Topic 4)

The students were divided into two groups; the first group developed representative tasks and the evaluation criteria (column B). The second group walked through the tasks, identified challenges (column C), and made recommendations to support those challenges (column D). Column E includes the course concepts to which the workflows are linked

This course-level innovation had the added benefit of contributing to the actual design of the vital signs monitoring device associated with the IMPALA project. Based on the tangible class discoveries, Dr. Vweza would document the errors the students encountered using the devices and their recommendations. Dr. Vweza then shared the documentation with the technical team of the IMPALA project to be considered in the next version of the device.

In addition to the cognitive walkthroughs with the IMPALA device, several other practical lessons used other devices available in the biomedical engineering lab at MUBAS as examples. For example, students would compare two different oxygen concentrators or infant incubators. Students would time different procedures using these devices and explore topics such as efficiency, satisfaction, and safety. While students compared and contrasted different on-market devices and noted shortfalls, these observations were not returned to designers. In summary, integrating the IMPALA project and other labs into the Human Factors course transformed the course into a hands-on experience in which students directly applied knowledge of course materials and reflected on the importance of the user's context.

### Reflection

Overall, the integration of the IMPALA project into the Human Factors course has given students at MUBAS a hands-on experience to apply their knowledge to the design of a medical device designed for use in Malawi, adding to a project whose initial design originated in a high-income country. The first aim of the project has been met, as students had the opportunity to critique and make suggested improvements for a device designed for their local context. The indicative content and course format is replicable in other countries and contexts, thus providing a template for others.

The second aim of the project was to instill an understanding of the importance of designing for the user’s context. Recognizing the increased importance of the context of the users [10], members of the class were able to provide a relevant perspective during the IMPALA design process. In particular, the members gave insights along several contextual dimensions [9]. Based on the instructor’s perspective, the students provided support for socio-cultural, infrastructural, and environmental factors and limited support for institutional and technological factors. As many biomedical engineers in Malawi support equipment repair in hospitals and clinics, they are possible future users of the device. Other important users for the IMPALA device include nurses and other healthcare workers, who have been included in the feedback process (but outside this course). As the IMPALA has evolved, students have engaged in several key steps in the design process, as recommended by Burleson et al. [11].

While direct assessment of this second aim has not been completed, the instructor and other faculty have informally noted the visible and successful integration of many human factors concepts into students’ final-year (i.e., capstone) design projects. Compared to projects before the addition of this course and against other departments without this course, the prototypes designed by BME students incorporated many features of user-centered design derived from the knowledge and skills gained from the Human Factors class. For example, one student team included color-coding for connectors as well as color-coded displays to indicate different operating states of a monitoring system used for the prevention of burns during monopolar electrosurgery. As a second example, another student team considered reprocessing concerns by reusing antimicrobial wood to build a prototype hospital bed capable of monitoring gram-level weight loss in cholera patients, thus considering the issue of availability of materials in resource-constrained settings.

Further, students were required to write a report on how the lessons from the Human Factors course and the IMPALA project applied to their final-year design project. While different students mentioned specific examples, the key theme that emerged was the suggestion to move the Human Factors class to an earlier year, rather than being offered during the same semester as their final-year projects. The students indicated that learning human factors earlier would help them apply those skills more effectively to their capstone project. Due to the considerable impact the Human Factors course has made, the course is being moved to third year, ahead of when the students do a mandatory internship and begin their final-year design project.

Regarding grading, 70% of the final course grade is the final exam (as MUBAS requires). Twenty percent of the course grade is related to the hands-on evaluation of medical devices, and 10% is the midterm exam. Frequent assignments related to hands-on learning included short reports on collected data on the human factors aspects of the device, in-class assessments on cognitive walkthroughs, and presentations/reports on the practical lab sessions. In the second semester of the 2020–2021 academic year, the course was taught by a different instructor and did not include the hands-on components. The first author taught the course in the second semesters of 2021–2022 and 2022–2023, including the hands-on elements. Given this shift in instructors, a direct comparison of the final exam grades would not be fruitful.

Direct assessment of the impact of this innovation will not be conducted, as removing the interactive modules to serve as a control would not support the education of the BME students at MUBAS. Rather, the proposed assessment would be to collect data from recent graduates who took the course to understand the impact of the course on their professional jobs. In particular, the authors would be interested in a survey (following Puerzer et al. [24]) focused on alumni perceived degree of importance and preparation on the topics of design and human factors, including how users are included in the design process, the role of cognitive walkthroughs, and how these factors impact design. Collecting and analyzing these data are planned for 2024.

#### Strengths and Weaknesses of Innovation and Implementation

At times, large class sizes (> 50) were difficult to manage during the hands-on components of the course. There were also difficulties in testing some of the software components of the IMPALA monitoring device, as moving through all aspects of the software was difficult; a simulation has been built for the device and Dr. Alick Vweza plans to use the simulator in upcoming ELE-HFM-521 lessons.

In terms of strengths, the collaboration between Dr. Alick Vweza and the IMPALA project harnessed the local expertise of MUBAS students to improve the design of a new patient monitoring system. Thus, the IMPALA project itself benefited from this collaboration. Second, the instructor believes that students benefited from applying the class concepts to construct feedback for the IMPALA, as well as interacting with their peers [22]. Additionally, the instructor was very pleased with their final exam and overall grades. As noted above, translating their knowledge to final-year design was seen as successful by faculty at MUBAS.

As instructors in high-income contexts consider medical device design for low-income contexts, it may be beneficial to create mutually supportive partnerships with local engineering schools for meaningful engagement, including human factors evaluation.

## Data Availability

N/A.

## Appendix

### Sample Lesson

The setting of the lesson is a standard lecture room with benches where students sit in groups. The lesson is allocated 110 min, with a 10-min break. The lesson is divided into 3 phases, namely:

1. Standard lecture that takes 50 min.
2. Practice on a real device that takes 30 min.
3. In-class assessment that takes 20 min.

### Topic: User Interface (UI) Requirements for Medical Devices

Lesson objectives: By the end of the lesson, the student should be able to the following:

1. Explain the importance of generating user interface requirements for medical devices.
2. Describe common pitfalls encountered when generating user interface requirements.
3. Carry out a cognitive walkthrough aimed at inferring which user interface requirements were used during the design of the medical device.
4. Develop high-quality user interface requirements for an existing medical device.

Lecture: Definitions, importance of generating UI requirements, common pitfalls encountered when generating UI requirements, example UI requirements for controls, displays, connectors and connections, and alarms.

Practice: The students are given the IMPALA device and are required to carry out a cognitive walkthrough with the aim of developing and understanding which UI requirements were developed for the design of the UI.

### Sample Cognitive Walkthrough Process for the IMPALA Monitor

1. Identify User Goals: Understand the primary goals users aim to achieve with the medical device UI. This should include tasks like setting parameters, inputting data, and accessing patient information.
2. Break Down Tasks: Divide the tasks into smaller steps to analyze each interaction point.
3. Step-by-Step Analysis: For each step, put yourself in the user's shoes and consider:
  - Visibility of Controls: Are necessary controls and options visible and easily accessible?
  - Feedback: Is there immediate feedback for user actions? For example, confirmation messages after data entry or successful completion of a task.
  - Error Prevention and Handling: Are error messages clear and helpful? Is there guidance on how to rectify errors?
  - Consistency: Is the UI consistent across different screens and functions?
  - Minimization of Memory Load: Are users required to remember information from previous screens or steps? Can information be presented in a way that reduces cognitive load?
  - Satisfying User Expectations: Does the UI behave in ways that users expect based on their experience with similar devices or interfaces?
  - Efficiency: Can tasks be completed efficiently without unnecessary steps or complications?
  - Safety Considerations: Are there safeguards in place to prevent errors that could potentially harm patients?
  - Accessibility: Is the UI designed to accommodate users with different levels of physical or cognitive abilities?

### Types of Observations

- Navigation: Observe how users navigate through different screens or sections of the UI. Look for any confusion or difficulty in finding specific functions.
- Data Entry: Pay attention to the ease of data entry and any potential errors users might encounter during this process.
- Alerts and Notifications: Note how alerts and notifications are presented to users and whether they effectively convey critical information without causing alarm or confusion.
- User Feedback: Gather feedback from users regarding their overall experience with the UI, including any frustrations or areas of improvement they may have encountered.

### Recommendations

- Simplify Complexity: Streamline the UI by removing unnecessary steps or options that may overwhelm users.
- Provide Clear Instructions: Ensure that instructions are clear and concise, guiding users through each step of the process.
- Enhance Feedback Mechanisms: Improve feedback mechanisms to provide users with real-time information on their actions and outcomes.
- Prioritize Safety: Implement additional safety features to minimize the risk of user error and ensure patient safety.
- Accessibility Improvements: Make adjustments to the UI to improve accessibility for users with disabilities or special needs.
- Iterative Testing: Continuously test and gather feedback from users to identify any further areas of improvement and refine the UI accordingly.

Sample Assessment: The students are grouped in teams of three. Each team is given the IMPALA device. Based on the cognitive walkthrough, each team is asked to develop at least three user interface (UI) requirements that were used to design each of the following parts of the IMPALA UI:

1. Controls
2. Displays
3. Connectors and connections
4. Alarms

## Code availability

N/A.
